# Network-Based Online Survey Exploring Self-Reported Depression Among University and College Students During the Early Days of the COVID-19 Outbreak

**DOI:** 10.3389/fpsyt.2021.658388

**Published:** 2021-05-13

**Authors:** Yurong Jing, Wantong Han, Yali Wang, Jiao Zhang, Wenzhe Qin, Xiang Jing, Aimin Niu, Lingzhong Xu

**Affiliations:** ^1^Centre for Health Management and Policy Research, School of Public Health, Cheeloo College of Medicine, Shandong University, Jinan, China; ^2^National Health Commission Key Laboratory of Health Economics and Policy Research, Cheeloo College of Medicine, Shandong University, Jinan, China; ^3^Shandong University Center for Health Economics Experiment and Public Policy Research, Jinan, China; ^4^Henan Provincial Center for Disease Control and Prevention, Zhengzhou, China; ^5^Yuncheng Central Hospital, Yuncheng, China; ^6^Department of Public Health, Shandong Provincial Hospital Affiliated to Shandong First Medical University, Jinan, China

**Keywords:** COVID-19, depression, university and college students, network-based survey, mental health

## Abstract

**Background:** The psychology of university and college students is immature, they are thus more likely to suffer from depression due to the COVID-19 pandemic. The present study aims to investigate the self-reported depression status of Chinese university and college students and explore its influencing factors.

**Methods:** We conducted a network-based online survey, and a total of 17,876 participants completed the questionnaire. Depression was measured by the Self-Rating Depression Scale (SDS). Univariate analysis and multivariate logistic analysis were performed to explore the influencing factors of self-reported depression symptoms.

**Results:** The proportion of self-reported depression symptoms, mild self-reported depression symptoms, and moderate to severe (M/S) self-reported depression symptoms was 65.2, 53.7, and 11.5%, respectively. The mean score of self-reported depression was 54.8 ± 9.0. Female, personality type of partial introversion, junior college educational level, “moderate” or “high” self-perceived risk of infection, “moderately” or “highly” impacted by the outbreak, and being eager to go back to school were risk factors for M/S self-reported depression symptoms (*p* < 0.05). While, “moderate” or “high” concern about the outbreak, “moderate” or “high” satisfaction with pandemic prevention and control measures, and having health literacy on communicable diseases were protective factors for M/S self-reported depression symptoms (*p* < 0.05).

**Conclusion:** The status of self-reported depression symptoms among university and college students was severer than expected, and the influencing factors were multifaceted. Government and school administrators should strengthen the dissemination of knowledge on disease prevention and control. Moreover, much attention should be paid to female and junior college students.

## Introduction

COVID-19 was first reported in Wuhan, China in early December 2019. The rapid development of the pandemic has attracted attention worldwide. The World Health Organization (WHO) declared this outbreak to be a public health emergency of international concern on January 31, 2020 (1). As of 24:00 on February 12, 2020, there were a total of 52,526 confirmed cases and 1,367 deaths in China (2), and confirmed cases had been reported in more than a dozen other countries. To contain the spread of the virus, Wuhan had been placed in lockdown, causing public's fears.

In addition to severely threatening people's physical health, the pandemic may also exacerbate their mental health disorders (3). Previous studies showed that abnormal psychological phenomenon was common during the SARS crisis, and depression was one of the most common mental disorders triggered by emerging infectious disease (EID) (4, 5). Recently, a Chinese study found that more than 50% of the 1,210 respondents from 194 cities reported moderate to severe (M/S) levels of psychological difficulties due to the COVID-19 outbreak, with about 16% suffering from symptoms of depression (6).

College students may be the most susceptible population for depression, since their psychological development is incomplete in the stage of transition from late adolescence to early adulthood (7–10). A meta-analysis involving 28,218 Chinese college students of 26 studies showed that 23.9% of college students had depression symptoms (11). Such a problem may become more serious during the pandemic, due to strict isolation measures, delays in school opening across the country, and lack of knowledge and skills on diseases prevention and control. A survey conducted in China found that 25.3% of 933 students from universities in Beijing and Wuhan had symptoms of depression during COVID-19 (12). Similarly, a survey of students from 85 different universities in Guangdong, China showed that 40.5% of 4,164 students were in a state of depression (13). Without early intervention, these depressive symptoms are more likely to develop into long-term depression, and lead to severe psychological disorders (14, 15).

College students' depression has been reported to be affected by many factors, including demographic factors (e.g., gender, personality type, education attainment, major, etc.) (16–18), and factors related to the pandemic (e.g., self-perceived risk of infection, knowledge and skills on diseases prevention and control, and satisfaction with government prevention and control measures, etc.) (19, 20). However, few studies investigated depression symptoms and its influencing factors among college students in China with a large sample during the early outbreak. Furthermore, most of the research related to mental health during the early COVID-19 outbreak focused on medical staff or patients (21, 22). Detecting early-onset mental health problems among college students may have many benefits, especially for campus health services and mental health policymaking (23, 24). Therefore, our study aims to conduct an online survey to investigate the self-reported depression status of Chinese university and college students, and to identify its influencing factors in a large sample size. Through this study, we hope to be able to provide appropriate management strategies to improve depressive symptoms for university and college students during the pandemic.

## Methods

### Participants

A convenience sampling method was used to collect data on February 20-22, 2020. University and college students were invited to participate in this survey via the internet using a self-administered questionnaire. The inclusion criteria were: (1) university and college students living in mainland China during the COVID-19 pandemic, (2) students able to complete the questionnaire on a cell phone or computer, and (3) informed consent. The exclusion criteria were: (1) students unable to use a computer or cell phone to complete the questionnaire, or (2) refusing to participate in the survey. A total of 18,294 questionnaires were collected, where there were 17,876 valid questionnaires after excluding invalid questionnaires that were incomplete or the answer time exceeded 20 min. The effective rate of the questionnaire was 97.7%. During the pandemic, joint prevention and control mechanisms and home quarantine were implemented in all areas of mainland China, and universities were closed during this period.

The protocol for this study was approved by the Ethical Committee of the Center for Health Management and Policy Research, Shandong University (No. ECCHMPSDU20201105), and all respondents provided informed consent.

### Measures

#### Social Demographic Characteristics

Social demographic characteristics included gender (male, female), ethnicity (Han, others), self-reported personality type (“partial introversion” means that the participants' personality is between introversion and extroversion, but more prone to introversion; “partial extroversion” corresponds to partial introversion; and “between partial introversion and partial extroversion” means that participants' personality is between partial introversion and partial extroversion), residence (city, town, and village), educational level (junior college, undergraduate, and master and above), and major (liberal arts, science and engineering, medical, and others).

#### Self-Reported Depression Symptoms

The Self-Rating Depression Scale (SDS) which was designed by Zung was adopted to assess participants' symptoms of depression during the past week (25). The SDS assesses depressive symptoms on a four-point scale ranging from “a little of the time” (value = 1) to “most of the time” (value = 4). The standard score is equal to the integer portion of 1.25 times the total score. The standard score of SDS is interpreted as: normal (≤52), mild (53–62), moderate (63–72), and severe (≥73) (26). Participants who had scores of 63 and above were characterized as having M/S self-reported depression symptoms. The Cronbach's α of the SDS in this study was 0.78.

#### Health Literacy on Communicable Diseases

Health literacy on communicable diseases was assessed using items from the China National Health Literacy Monitoring Questionnaire, which was compiled by the China Health Education Center in 2016 (27). Participants who had total scores of 5.6 and above were judged to have health literacy on communicable diseases (28).

#### Measurement of Other Variables

Other personal information was also collected in this study, including self-perceived risk of infection (high, moderate, and low), impacted by the outbreak (highly, moderately, and lowly), concern about the outbreak (high, moderate, and low), satisfaction with pandemic prevention and control measures (high, moderate, and low), and being eager to go back to school (no, yes, and uncertain).

### Investigation Method

The electronic “Questionnaire Star” tool (Changsha Ranxing Science and Technology, China, https://www.wjx.cn/) was used as the survey tool, and the information was collected through sending anonymous survey links by a member of the research team to WeChat groups. Participants were required to complete the questionnaire within 20 min, and each IP could only be filled in once. In addition, the questionnaire could only be filled out through WeChat, and one WeChat account can only be filled out once. As a professional online survey platform, which can be used for questionnaire survey, evaluation, voting, and other purposes, the “Questionnaire Star” has strengths in being fast, at low cost, and easy to learn and use (29). It has been applied in some investigations related to COVID-19 (19, 30).

### Statistical Analysis

All statistical analyses were performed using the IBM SPSS Statistical Software (version 22.0). First, frequency, percentage, mean, and standard deviations were calculated for all variables. Second, the non-parametric Mann-Whitney U-test and Kruskal-Wallis test were applied to compare the severity of self-reported depression symptoms among different groups. Third, a multivariate logistic regression analysis was performed to identify influencing factors for M/S self-reported depression symptoms. A *p*-value of <0.05 was considered to indicate statistical significance.

## Results

### Characteristics of Participants

The demographics of the study population are shown in Table 1. Among the samples of 17,876 responding participants, most of the participants were female (71.7%), Han (88.7%), personality type of between partial introversion and partial extroversion (61.2%), living in a village (51.0%), educational level of junior college students (51.6%), and medical students (61.4%).

**Table 1:** Demographic characteristics of the university and college students by self-reported depression status.

**Characteristic**			**Depression symptoms**			**Statistics**
	**Total (%)**	**M ± SD**	**Normal (%)**	**Mild (%)**	**M/S (%)**	
Observations	17,876 (100.0)	54.8 ± 9.0	6,216 (34.8)	9,603 (53.7)	2,057 (11.5)	
**Gender**						−2.86^a^^**^
Male	5,058 (28.3)	55.1 ± 9.1	1,591 (31.5)	2,976 (58.8)	491 (9.7)	
Female	12,818 (71.7)	54.6 ± 8.9	4,625 (36.1)	6,627 (51.7)	1,566 (12.2)	
**Ethnicity**						−11.01^a^^***^
Han	15,850 (88.7)	54.5 ± 9.1	5,748 (36.3)	8,327 (52.5)	1,775 (11.2)	
Others	2,026 (11.3)	57.1 ± 8.0	468 (23.1)	1,276 (63.0)	282 (13.9)	
**Personality type**						6.41^b^^*^
Partial introversion	3,882 (21.7)	55.2 ± 8.6	1,304 (33.6)	2,085 (53.7)	493 (12.7)	
Partial extroversion	3,060 (17.1)	54.4 ± 9.2	1,076 (35.2)	1,658 (54.2)	326 (10.7)	
Between partial introversion and partial extroversion	10,934 (61.2)	54.7 ± 9.1	3,836 (35.1)	5,860 (53.6)	1,238 (11.3)	
**Residence**						3.68^b^
City	4,239 (23.7)	54.5 ± 9.4	1,547 (36.5)	2,188 (51.6)	504 (11.9)	
Town	4,524 (25.3)	54.9 ± 9.1	1,559 (34.5)	2,412 (53.3)	553 (12.2)	
Village	9,113 (51.0)	54.9 ± 8.7	3,110 (34.1)	5,003 (54.9)	1,000 (11.0)	
**Education**						711.49^b^^***^
Junior college	9,230 (51.6)	56.6 ± 8.3	2,356 (25.5)	5,560 (60.2)	1,314 (14.2)	
Undergraduate	8,185 (45.8)	52.9 ± 9.2	3,630 (44.3)	3,859 (47.1)	696 (8.5)	
Master and above	461 (2.6)	51.5 ± 10.5	230 (49.9)	184 (39.9)	47 (10.2)	
**Major**						180.66^b^^***^
Liberal arts	1,664 (9.3)	54.2 ± 8.9	646 (38.8)	843 (50.7)	175 (10.5)	
Science and engineering	3,461 (19.4)	53.0 ± 9.1	1,490 (43.1)	1,696 (49.0)	275 (7.9)	
Medical	10,972 (61.4)	55.3 ± 9.0	3,538 (32.2)	6,044 (55.1)	1,390 (12.7)	
Others	1,779 (10.0)	55.5 ± 8.5	542 (30.5)	1,020 (57.3)	217 (12.2)	
**Self-perceived risk of infection**						94.04^b^^***^
Low	15,206 (85.1)	54.5 ± 9.1	5,469 (36.0)	8,102 (53.3)	1,635 (10.7)	
Moderate	2,398 (13.4)	56.3 ± 8.3	680 (28.4)	1,347 (56.2)	371 (15.5)	
High	272 (1.5)	57.4 ± 8.7	67 (24.6)	154 (56.6)	51 (18.8)	
**Impacted by the outbreak**						148.48^b^^***^
Lowly	4,090 (22.9)	53.5 ± 9.6	1,608 (39.3)	2,169 (53.0)	313 (7.7)	
Moderately	7,294 (40.8)	54.6 ± 8.7	2,584 (35.4)	3,960 (54.3)	750 (10.3)	
Highly	6,492 (36.3)	55.8 ± 8.7	2,024 (31.2)	3,474 (53.5)	994 (15.3)	
**Concern about the outbreak**						14.93^b^^**^
Low	422 (2.4)	56.3 ± 8.6	115 (27.3)	241 (57.1)	66 (15.6)	
Moderate	9,259 (51.8)	54.9 ± 8.7	3,209 (34.7)	4,985 (53.8)	1,065 (11.5)	
High	8,195 (45.8)	54.5 ± 9.4	2,892 (35.3)	4,377 (53.4)	926 (11.3)	
**Satisfaction with pandemic prevention and control measures**			69.45^b^^***^
Low	284 (1.6)	58.2 ± 9.0	61 (21.5)	154 (54.2)	69 (24.3)	
Moderate	4,632 (25.9)	55.2 ± 8.8	1,551 (33.5)	2,417 (52.2)	664 (14.3)	
High	12,960 (72.5)	54.5 ± 9.0	4,604 (35.5)	7,032 (54.3)	1,324 (10.2)	
**Eager to go back to school**						15.21^b^^***^
Yes	6,052 (33.9)	55.0 ± 9.0	2,051 (33.9)	3,194 (52.8)	807 (13.3)	
No	7,035 (39.4)	54.7 ± 9.0	2,432 (34.6)	3,864 (54.9)	739 (10.5)	
Uncertain	4,789 (26.8)	54.5 ± 8.9	1,733 (36.2)	2,545 (53.1)	511 (10.7)	
**Health literacy on communicable diseases**						−12.23^a^^***^
No	14,488 (81.0)	55.2 ± 8.8	4,715 (32.5)	8,036 (55.5)	1,737 (12.0)	
Yes	3,388 (19.0)	52.9 ± 9.5	1,501 (44.3)	1,567 (46.3)	320 (9.4)	

* *p < 0.05*,

** *p < 0.01*,

*** *p < 0.001*.

*M, mean; SD, standard deviation*.

a *Mann-Whitney U-test*.

b *Kruskal-Wallis test*.

### Status of Self-Reported Depression Symptoms by Subgroup

Of the 17,876 students, the mean score of self-reported depression was 54.8 ± 9.0, and the proportion of self-reported depression symptoms corresponding to normal, mild, and M/S was 34.8, 53.7, and 11.5%, respectively. The individuals who were female, minorities, had a personality type of partial introversion, educational level of junior college, majoring in a medical field, “high” self-perceived risk of infection, “highly” impacted by the outbreak, “low” concern about the outbreak, “low” satisfaction with pandemic prevention and control measures, being eager to go back to school, or not having health literacy on communicable diseases were more inclined to severe self-reported depression symptoms (*p* < 0.05). Moreover, residence had no significant effect on self-reported depression symptoms (*p* > 0.05) (Table 1).

### Factors Influenced With Self-Reported Depression Symptoms

Results of multivariate logistic regression analysis of factors associated with M/S self-reported depression symptoms during the early COVID-19 outbreak are presented in Table 2. The results indicated that being female (OR = 1.289, *p* < 0.001), having a “moderate” or “high” self-perceived risk of infection (OR = 1.338, *p* < 0.001; OR = 1.443, *p* = 0.024, respectively), were “moderately” or “highly” impacted by the outbreak (OR = 1.324, *p* < 0.001; OR = 2.048, *p* < 0.001, respectively), and being eager to go back to school (OR = 1.218, *p* < 0.001) were risk factors for M/S self-reported depression symptoms. While, personality type of “partial extroversion” or “between partial introversion and partial extroversion” (OR = 0.771, *p* = 0.001; OR = 0.862, *p* = 0.010, respectively), educational level of “undergraduate” or “master and above” (OR = 0.533, *p* < 0.001; OR = 0.635, *p* = 0.005, respectively), “moderate” or “high” concern about the outbreak (OR = 0.734, *p* = 0.029; OR = 0.692, *p* = 0.011, respectively), “moderate” or “high” satisfaction with pandemic prevention and control measures (OR = 0.586, *p* < 0.001; OR = 0.394, *p* < 0.001, respectively), and having health literacy on communicable diseases (OR = 0.744, *p* < 0.001) were protective factors for M/S self-reported depression symptoms.

**Table 2:** Multivariate logistic regression analysis of factors influenced with M/S self-reported depression symptoms.

**Characteristic**	**β**	**SE**	**OR**	**95% CI**	***p*-value**
**Gender (ref: male)**					
Female	0.254	0.058	1.289	(1.151, 1.443)	<0.001
**Personality type (ref: partial introversion)**					
Partial extroversion	−0.260	0.078	0.771	(0.662, 0.897)	0.001
Between partial introversion and partial extroversion	−0.149	0.058	0.862	(0.769, 0.965)	0.010
**Education status (ref: junior college)**					
Undergraduate	−0.630	0.062	0.533	(0.472, 0.601)	<0.001
Master and above	−0.455	0.160	0.635	(0.463, 0.869)	0.005
**Self-perceived risk of infection (ref: low)**					
Moderate	0.291	0.065	1.338	(1.179, 1.519)	<0.001
High	0.366	0.163	1.443	(1.049, 1.984)	0.024
**Impacted by the outbreak (ref: lowly)**					
Moderately	0.281	0.072	1.324	(1.150, 1.524)	<0.001
Highly	0.717	0.071	2.048	(1.781, 2.356)	<0.001
**Concern about the outbreak (ref: low)**					
Moderate	−0.309	0.142	0.734	(0.555, 0.969)	0.029
High	−0.368	0.144	0.692	(0.522, 0.918)	0.011
**Satisfaction with pandemic prevention and control measures (ref: low)**					
Moderate	−0.534	0.149	0.586	(0.438, 0.785)	<0.001
High	−0.933	0.146	0.394	(0.296, 0.524)	<0.001
**Eager to go back to school (ref: no)**					
Yes	0.198	0.056	1.218	(1.091, 1.361)	<0.001
Uncertain	−0.032	0.062	0.968	(0.858, 1.093)	0.604
**Health literacy on communicable diseases (ref: no)**					
Yes	−0.296	0.066	0.744	(0.654, 0.846)	<0.001

*CI, confidence interval*.

## Discussion

In our study, the proportion of self-reported depression symptoms was 65.2%, where mild and M/S accounted for 53.7 and 11.5%, respectively. Our finding is higher than that of several online surveys conducted in the general population during the COVID-19 pandemic (17.2–37.1%) (6, 31, 32), and it is also higher than a study conducted in the UK among college students (46.5%) (33). Moreover, the mean score of self-reported depression was 54.8 ± 9.0, which is higher than the Chinese norm (41.88 ± 10.57) and a study conducted in Shandong, China (42.47 ± 8.61) (*p* < 0.05) (34). Possible explanations for the higher proportion and mean score of self-reported depression symptoms in our study are that Chinese students are more likely to suffer from depressive symptoms because of fear caused by widespread media coverage on an increasing number of confirmed and suspected cases in China, lack of relevant knowledge and skills on prevention and control, and shortage of specific treatment. However, our result is lower than the study in Guangdong, China (79.9%) (35), which was conducted among 488 medical students using the Depression Status Inventory (DSI). It is likely that our study involved other majors' students besides medical students and we used a different measurement scale. Previous studies have proven that compared to students in other majors, medical students were susceptible to greater levels of depression due to the heavy burden of study, absence of any leisure activities, and exposure to death and suffering (36, 37).

Consistent with previous studies, female students self-reported more severe depression symptoms than males (38, 39). One possible reason is that female students are more emotional and sensitive to severe events than males (40). Likewise, a study during the period of SARS showed that female students' understanding of SARS was more perceptual, and they lacked a rational perspective on the infection and treatment of SARS (41).

Compared with undergraduates and students with a higher educational level, junior college students were more likely to suffer from M/S self-reported depression symptoms due in part to lacking knowledge and skills on disease prevention and control. They may also experience more stress because of low academic qualification when they are looking for a job. Therefore, they are prone to have depressive symptoms and psychological distress during the pandemic. In addition, higher concern about the outbreak and having health literacy were protective factors for M/S self-reported depression symptoms. Students who are more concerned about the pandemic may have a higher understanding of the pandemic. Previous research among college students during SARS showed that students with higher cognition had a lower risk of depression symptoms (42, 43).

Our study showed that students who were eager to go back to school or those highly impacted by the outbreak were more likely to suffer from M/S self-reported depression symptoms. To contain the pandemic, the education department postponed school opening. Staying at home for a long time may increase the risk for self-reported depression symptoms. Their lifestyle and study plan may be correspondingly changed, further increasing the risk for self-reported depression symptoms among college students. Our study also found that partially introverted students had a higher risk of suffering from more severe self-reported depression symptoms, which is consistent with most previous studies (17, 44, 45). Introversion is linked to decreased help-seeking behavior, and introverts are thus more likely to turn inward to cope with negative emotions (46, 47). Over time, the negative impacts caused by the pandemic may exacerbate their depression.

Another finding in our study was that students who had lower satisfaction with prevention and control measures, or those who had higher self-perceived risk of infection were more prone to have self-reported M/S depression symptoms. Similarly, a previous study showed that people with confidence in government measures were less likely to have emotionally distressful responses during the avian influenza epidemic (20). Due to the lack of understanding the pandemic, students who perceived higher risk of infection were more likely to be affected by the pandemic, resulting in a higher status of self-reported depression.

Despite these advantages, there were several limitations in our study. Firstly, our data were self-reported via an online network, and the SDS scale was used to detect and screen depression symptoms, which may be less accurate than rating from a clinical psychologist or psychiatrist. Secondly, this is a cross-sectional study; therefore, associations cannot be viewed as causal relationships. Future research should consider a longitudinal design to follow up on the change in the students' psychological status to provide necessary support. Thirdly, convenience sampling was used in our study, which was not based on a random selection of the sample; thus, the study population did not reflect the actual pattern of the general population. Finally, due to the limitation of an online survey, it was impossible to investigate more factors in our study.

## Conclusion

In this study, university and college students had a higher proportion of self-reported depression symptoms than expected. The influencing factors of self-reported depression symptoms were multifaceted, including socio-demographic factors and those related to the pandemic. Governments should provide more disease prevention and control services to improve knowledge and skills on disease prevention for college students, and boost their confidence in fighting against the pandemic. School managers should also pay more attention to female and junior college students in health education and promotion.

## Data Availability Statement

The raw data supporting the conclusions of this article will be made available by the authors, without undue reservation.

## Author Contributions

YJ and WH performed material preparation and data collection. YJ, WH, YW, and XJ performed statistical analysis. YJ writing the manuscript. LX, YW, JZ, and WQ helped revise the manuscript. LX and AN directed the study. All authors have contributed significantly to the manuscript, read, and approved the final manuscript.

## Conflict of Interest

The authors declare that the research was conducted in the absence of any commercial or financial relationships that could be construed as a potential conflict of interest.
